# Netherton Syndrome With Trichorrhexis Invaginata “Bamboo Hair” Under Dermoscopy: Case Images

**DOI:** 10.1002/ccr3.71755

**Published:** 2025-12-29

**Authors:** Khalid Nabil Nagshabandi, Abdulaziz Alsalhi

**Affiliations:** ^1^ Department of Dermatology, College of Medicine King Saud University Riyadh Saudi Arabia; ^2^ Department of Medicine King Abdullah Bin Abdulaziz University Hospital Riyadh Saudi Arabia

**Keywords:** bamboo hair, dermoscopy, genodermatosis, golf tee, Netherton syndrome, trichorrhexis invaginata, trichorrhexis nodosa, trichoscopy

## Abstract

Early recognition of Netherton syndrome in children can be prompted by dermoscopic detection of trichorrhexis invaginata (“bamboo hair”) together with ichthyosis linearis circumflexa. Dermoscopy of eyebrow hairs is a simple, noninvasive clue that expedites diagnosis, counseling, and supportive care while genetic testing is pursued.

## 1 Introduction

Netherton syndrome (NS), first described by Comèl in 1949 and later named by Netherton in 1958, is a rare autosomal recessive genodermatosis clinically characterized by ichthyosis linearis circumflexa (ILC), atopic features (a predisposition to atopic manifestations including eczema, allergies, and elevated serum IgE levels), and abnormalities in hair shaft structure known as trichorrhexis invaginata (TI) (also termed “bamboo hair”) [1]. NS has an incidence of 1 out of 200,000 new live births [1]. Genetic investigations have linked NS to a mutation in the serine protease inhibitor (SPINK5) gene on chromosome 5q31‐32, which encodes the lymphoepithelial Kazal type‐related inhibitor (LEKTI), which is crucial for maintaining proper skin and hair function. Defective expression of LEKTI is prevalent in NS patients, causing premature desquamation of the stratum corneum leading to subsequent skin impairment and hair shaft abnormalities [1, 2].

## 2 Case Presentation

**FIGURE 1:**
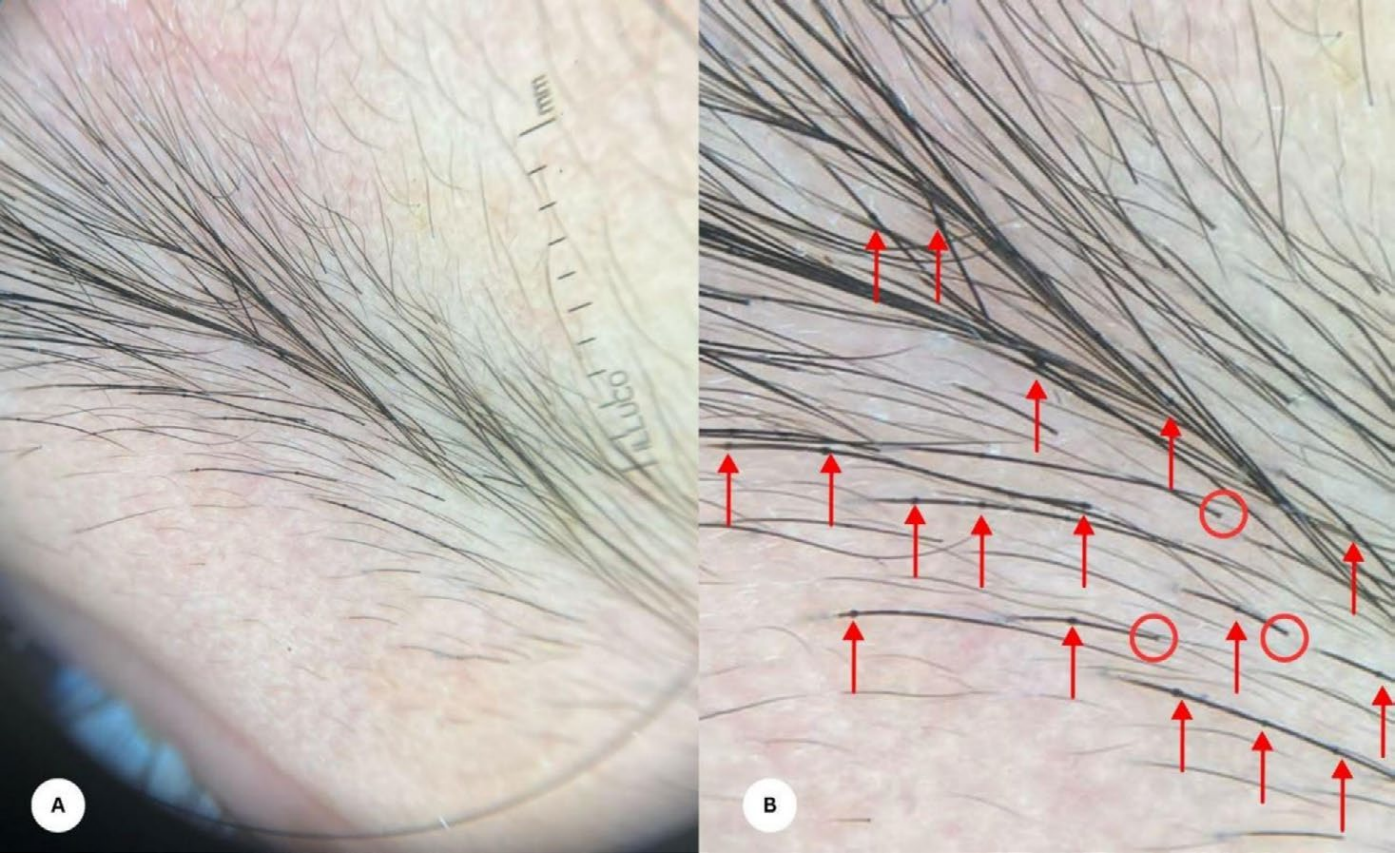
(A, B) Trichoscopic exam of the eyebrow hair revealed the characteristic trichorrhexis invaginata or bamboo hair sign (red arrows), and golf tee sign (red circles), which is consistent with the diagnosis of Netherton syndrome.

A 5‐year‐old girl, known to have asthma, presented to the outpatient dermatology clinic with recurrent pruritic eruption since birth that partially improves with topical mometasone, along with parents' complaints that her hair is always short and never needed a haircut since birth. Cutaneous examination revealed evident presentation of erythematous, serpiginous plaques with double‐edged scales across the limbs showcasing a linear and tramline‐like pattern, characteristic lesion indicative of ichthyosis linearis circumflexa, eczematous patches on the face. Examination of the scalp revealed sparse, lustrous, brittle hair. Dermoscopic examination of the eyebrow hair showed findings consistent with trichorrhexis invaginata or “bamboo hair”, and golf tee signs (Figure 1). A skin punch biopsy was obtained from the lesions, and histopathological evaluation showed signs of hyperkeratosis, acanthosis, and spongiosis, with a dermal lymphocytic infiltration. Clinical, dermoscopic, and biopsy findings all suggest the diagnosis of NS. The patient was managed with topical corticosteroids and frequent skin moisturization. The family was advised to seek genetic testing for confirmation.

Trichorrhexis invaginata “Bamboo Hair” represents the hallmark hair shaft anomaly observed under dermoscopy/trichoscopy exam in NS. Affected hair shafts invaginate from the distal portion into the proximal portion due to the softness of the cortex in the keratogenous zone, resembling bamboo nodes and mimicking “ball‐and‐socket‐like appearance”. To confirm the diagnosis of NS, TI must be detected in at least one hair [1]. Trichorrhexis Nodosa (weak points along the hair shaft, leading to their breakage), though non‐specific, is the most common hair shaft sign seen in NS [2, 3]. Pili torti (twisting of the hair along its axis) is another hair shaft abnormality that can be seen in NS, which contributes to the overall fragility and abnormal appearance of the hair [3]. Additionally, “matchstick” (bulbous or enlarged hair ends) and “golf tee” (distal end of hair shaft wider than proximal) hairs can also present as distinct dermoscopic features in individuals with NS [1, 2, 3]. There is no particular curative modality for NS. Management is typically symptomatic and customized for each case. Topical corticosteroids, calcineurin inhibitors, topical and systemic retinoids, narrowband UVB phototherapy, and immunosuppressant drugs are all viable treatment choices with varying degrees of success. New insights with topical pimecrolimus 1% cream revealed little systemic absorption and a satisfactory safety and effectiveness profile [1, 2, 3].

## Author Contributions

Conceptualization: Khalid Nabil Nagshabandi; Abdulaziz Alsalhi. Clinical investigation and patient care: Khalid Nabil Nagshabandi; Abdulaziz Alsalhi. Data curation and resources: Khalid Nabil Nagshabandi; Abdulaziz Alsalhi. Visualization (image acquisition/annotations/figure prep): Khalid Nabil Nagshabandi. Literature review: Khalid Nabil Nagshabandi; Abdulaziz Alsalhi. Writing – original draft: Khalid Nabil Nagshabandi. Writing – review and editing: Khalid Nabil Nagshabandi; Abdulaziz Alsalhi. Guarantor: Khalid Nabil Nagshabandi.

## Funding

The authors have nothing to report.

## Ethics Statement

Ethical approval is not required for this study in accordance with local or national guidelines.

## Consent

Written informed consent was obtained from the patient for publication of the details of their medical case and any accompanying images.

## Conflicts of Interest

The authors declare no conflicts of interest.

## Data Availability

All data generated or analyzed during this study are included in this article. Further enquiries can be directed to the corresponding author.

## References

[1] M. E. Herz‐Ruelas , S. Chavez‐Alvarez , J. I. Garza‐Chapa , J. Ocampo‐Candiani , V. A. Cab‐Morales , and D. E. Kubelis‐López , “Netherton Syndrome: Case Report and Review of the Literature,” Skin Appendage Disorders 7, no. 5 (2021): 346–350, 10.1159/000514699. 34604321 PMC8436607

[2] M. J. Bittencourt , E. R. Moure , O. T. Pies , A. D. Mendes , M. M. Deprá , and A. L. Mello , “Trichoscopy as a Diagnostic Tool in Trichorrhexis Invaginata and Netherton Syndrome,” Anais Brasileiros de Dermatologia 90, no. 1 (2015): 114–116, 10.1590/abd1806-4841.20153011. 25672309 PMC4323708

[3] H. M. K. Saleem , M. F. Shahid , A. Shahbaz , A. Sohail , M. A. Shahid , and I. Sachmechi , “Netherton Syndrome: A Case Report and Review of Literature,” Cureus 10, no. 30 (2018): e3070, 10.7759/cureus.3070. 30280066 PMC6166913

